# Utilising a collective case study system theory mixed methods approach: a rural health example

**DOI:** 10.1186/1471-2288-14-94

**Published:** 2014-07-28

**Authors:** Robyn Adams, Anne Jones, Sophie Lefmann, Lorraine Sheppard

**Affiliations:** 1 Discipline of Physiotherapy, James Cook University, Townsville, Australia; 2 Discipline of Physiotherapy, University of South Australia, Adelaide, Australia

**Keywords:** Case study, Health service, Rural, Systems theory

## Abstract

**Background:**

Insight into local health service provision in rural communities is limited in the literature. The dominant workforce focus in the rural health literature, while revealing issues of shortage of maldistribution, does not describe service provision in rural towns. Similarly aggregation of data tends to render local health service provision virtually invisible. This paper describes a methodology to explore specific aspects of rural health service provision with an initial focus on understanding rurality as it pertains to rural physiotherapy service provision.

**Method:**

A system theory-case study heuristic combined with a sequential mixed methods approach to provide a framework for both quantitative and qualitative exploration across sites. Stakeholder perspectives were obtained through surveys and in depth interviews. The investigation site was a large area of one Australian state with a mix of rural, regional and remote communities.

**Results:**

39 surveys were received from 11 locations within the investigation site and 19 in depth interviews were conducted. Stakeholder perspectives of rurality and workforce numbers informed the development of six case types relevant to the exploration of rural physiotherapy service provision. Participant perspective of rurality often differed with the geographical classification of their location. The numbers of onsite colleagues and local access to health services contributed to participant perceptions of rurality.

**Conclusions:**

The complexity of understanding the concept of rurality was revealed by interview participants when providing their perspectives about rural physiotherapy service provision. Dual measures, such as rurality and workforce numbers, provide more relevant differentiation of sites to explore specific services, such rural physiotherapy service provision, than single measure of rurality as defined by geographic classification. The system theory-case study heuristic supports both qualitative and quantitative exploration in rural health services research.

## Background

Aggregation of health service and workforce data can render local rural health service issues virtually invisible. As data is collated from local, regional, state and national data sets, visibility of local service availability and accessibility diminish with each level of data collation. Obtaining insight into local health service provision beyond the data requires a different approach: a framework that can provide insight into service delivery and variability in a way existing statistical data does not. The aim of this paper is to describe a methodology to explore specific aspects of rural health service provision with an initial focus on understanding rurality as it pertains to rural physiotherapy service provision. There is little identified literature which describes decision making in rural physiotherapy service provision. No known study has identified influencing factors and what processes are involved in understanding what physiotherapy services to provide in rural locations. Thus there was a need to identify what methodology to use to investigate this topic.

### Defining place

The concept of *relational place* has been suggested as useful for exploration of the specifics of rural communities and the impact of context [1]*.* This concept of place is informed by the relational understanding of space and place advocated by Cummins et al. [2] and ‘the event of place’ referred to by Massey [3]. The relational view of place includes elements such as nodes of networks, separated by social relational distance with populations of individuals who are mobile both daily and over their life course [2]. Definitions of the area of the ‘place’ are relatively fluid, recognising dynamic characteristics of place such as ‘declining’ or ‘advancing’ in contrast to conventional characteristics such as ‘deprived’ or ‘affluent’, which are fixed at points in time [2].

**Figure 1:**
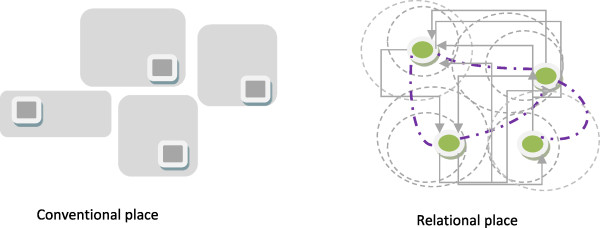
Conceptual representation of conventional and relational place.

Distinguishing attributes of cultural divisions, services and infrastructure is important in understanding health service provision. The conventional view of place is one of ‘culturally neutral territorial division, infrastructure and services’ where services are described ‘in terms of fixed locations often providing for territorial jurisdiction and distance decay models’ [2], p. 1827. This contrasts with the relational view of place which sees ‘territorial divisions, services and infrastructure imbued with social power relations and cultural meaning’ [2], p. 1827. The conventional view of place as spaces with fixed geographical boundaries and services described in terms of fixed locations [2] is different to the relational view of ‘nodes of networks’; ‘constellations of connections’ [3] and complex circuitry with a multiplicity of linkages and feedback loops [4]. Conceptual representation of conventional and relational space is provided Figure 1. Thus when exploring rural health service provision an understanding of place and the definition used to identify place will impact on the results of the study. Although a health region has defined boundaries seen in the conventional view of place, it is the relational view of place which shows the interactions between and across boundaries which really matches rural health service provision. The multiple linkages and complex nature of health services influences the methodological approach to researching rural health service provision and decision making.

## Methods

### Mixed methods

Combining qualitative and quantitative methods provides possibilities for health researchers to grapple with the complexity of health [5], and the factors that influence both health care and health service provision. A mixed methods approach allows quantitative data obtained from surveys and health service data to be combined with qualitative data to better inform decisions about health service delivery. A qualitative paradigm supports exploration of issues and factors at local, state and national levels that influence local of health service provision. Obtaining stakeholder perspectives on specific aspects of a health service provides insight into local health service provision beyond the quantitative data. Approaches used within qualitative research enable participants to reveal their thoughts and perceptions within their context [6]. This is highly relevant to health service research because it supports exploration of the perspectives of multiple stakeholders across different geographical settings and different sized and types of services. Variability and diversity are characteristic of rural Australia [7], and combine with the unique demography of rural and remote Australia as key determinants of health problems and health service needs [8]. Thus, a robust methodological approach should consider variability and diversity of different sites, to be consistent with the dynamic characteristics of place.

Adopting a sequential mixed method approach provides a framework for health researchers to investigate an issue of interest in complex organisations by providing structure for obtaining data from multiple sources. A preliminary quantitative component, such as a survey, can precede and guide the main qualitative data collection by informing purposive sampling and establishing preliminary results for further in-depth exploration [9]. Use of an initial survey, for example, allows the researcher to obtain the perspectives of a broader range of stakeholders than may be feasible if only a qualitative approach is adopted. By analysing the data from the quantitative component groups of interest can be identified. Use of stratified purposive sampling then permits the exploration of ideas by these groups [10] in the qualitative component. These groups may include service providers, their colleagues, managers, key decision-makers or consumers. This supports a key intent of sampling within qualitative research, which is the selection of information rich cases [11]. The perspectives of individuals enables the researcher to look for complexity of views and also to address processes of interaction among individuals [12]. Researchers can focus on the specific context in which people live and work [12] which is important when seeking to understand the setting in which health services are delivered.

### Case study

Case study design supports the use of multiple data sources. It is appropriate where the research aim is to explore contextual or complex multivariate conditions and not just isolated variables [13]. An instrumental case study approach the issue or factor is the focus of the study rather than the case [14]. Organisational complexity and contextual factors such rurality and service settings are important factors in health service delivery. Adopting an instrumental case study approach is suitable therefore as it allows a focus on the issue of interest across sites. Stake refers to the study of more than one case as collective case studies, each of which is an instrumental study linked by coordination between individual studies [14]. Collective case study design [14] provides a structure to gain insight into the issue of interest across settings as it allows comparison within and between cases [15]. Thus the use of instrumental collective case studies is useful for identifying and studying factors that affect service level decision making in rural health.

### Systems theory

A systems-focused approach is recommended to articulate interdependent components that contribute to or compromise the effectiveness of health care interventions or programs. It provides insight into the questions of ‘why’ , ‘how’ and the ‘what’ of contexts [16]. ‘The parts do not have to be working well, the purpose may be irrational, but it is a system none the less’ [14], p. 2. The boundary of a system need not correspond with recognised departmental, institutional or other physical boundaries [11]. The exchange of inputs and outputs across a boundary indicate boundary permeability. An interaction with the environment is characteristic of systems generally and more specifically of open systems [17,18]. Although boundaries may be clearly defined, they are subject to interaction and influences external to the system. Within an interpretive paradigm it is “acknowledged that a ‘system’ is not a concrete thing but an abstract concept that constitutes particular relationships that can be actualised in a number of ways” [18], p. 128. Perceiving one aspect or specific issue as a system within a more broadly conceived organisation can generate both a new representation of the issue and variety in the way the issue is thought about [11]. Choosing to think of a health care organisation as if it were a system is a useful construct and one that is not new in its application to health and health care [19]. Studies of emergency department physiotherapy [20] and health promoting environments in a university [21] for example, have described sets of interrelated elements which, when viewed together, form a ‘whole’. What is new is applying it to service level decision making in the context of rural health.

### Combining case study and systems theory

The constructs within the relational view of place suggest the relevance of both case study and systems theory. Both methodologies have been used in health and health care research [21-24]. Combining systems theory and case study methodology offers the opportunity for in-depth exploration as well as comparative analysis between cases in the context of the system [20]. The combination provides a way of conceptualising complex issues for exploration, and as such has been considered an heuristic model [25]. This model provides a structure to explore local health service provision whilst recognising the flows and linkages that occur within a relational concept of place. The systems theory-case study heuristic, as part of a framework for this study, acknowledges that a case is also a ‘bounded system’ [14], p. 2. This notion assists by drawing attention to it as an object rather than a process.

Organisational issues, including how and why health care services are provided, requires recognition of a range of influencing factors. Also important is an understanding of the impact of external factors, such as national and state health policies, on health services provided by an organisation.

**Figure 2:**
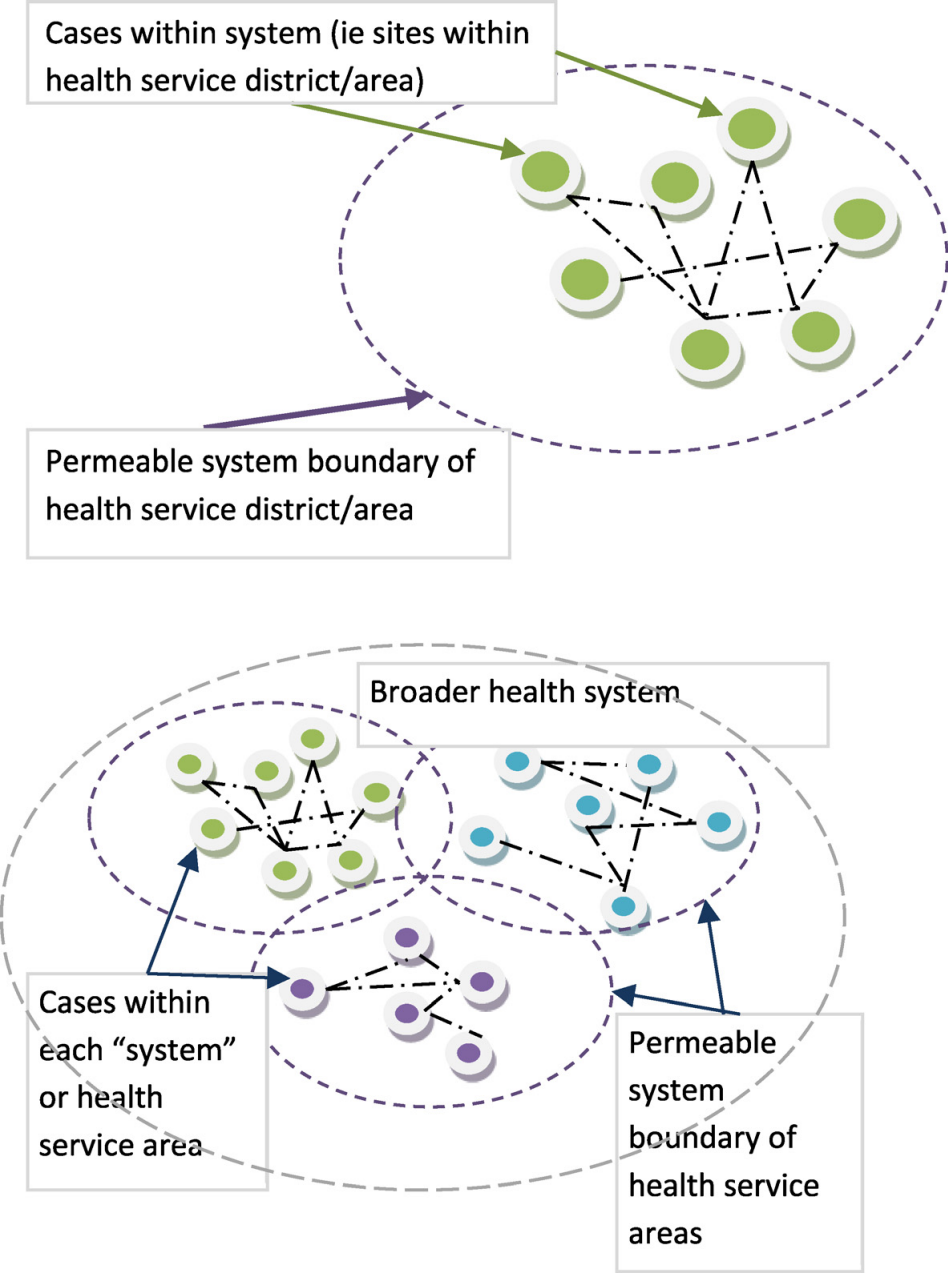
Demonstration of cases within a health system.

Adoption of both a collective instrumental case study design and a systems approach supports the focus on the issue of interest and holistic exploration [24]. In this study of rural physiotherapy service provision the defined system may include tangible and intangible elements within a broader organisation that are connected to form a system. The application of both case study and a system theory approach supports consideration of a single issue across multiple sites and contextual variations of place. Each of these individual cases exists within larger systems with interactions between cases and is influenced beyond both the boundaries of the single case and the network boundaries (Figure 2).

The systems theory-case study heuristic supports the use of both qualitative and quantitative approaches and a priori–sequence model [5] guides the practical integration of both approaches. As part of a larger study seeking to understand how decisions are made about rural physiotherapy service provision, important first steps were to understand participant’s perspectives of rurality and to develop cases for exploration of decision making about physiotherapy service provision.

### Context of rurality

Due to the expressed limitations of the application of geographic classifications to health care [26], the researchers focussed on Participant Perspectives of Rurality(PPR) which were compared with geographical classifications. Many definitions and classifications are used to describe or differentiate rural, regional and remote settings [27,28]. The Australian Standard Geographical Classification Remoteness Areas [ASGC- RA] is recommended by the Australian Institute of Health and Welfare (AIHW) [29]. Other Australian classifications include Access/Remoteness Index for Australia [ARIA] and Rural, Remote & Metropolitan Areas Classification [RRMA]. As classifications are based on factors such as distance to service centres, population size or density, they may not take into account other contextual factors or accessibility of specific health services such as physiotherapy. Different definitions can lead to different classifications and, in terms of program funding for instance, may alter a community or individual’s entitlement eligibility. The notion of developing a suite of measures [26,30] is important in the exploration of specific issues of health and health service provision in rural settings. The index of access to primary care is one recently described measure [30]. The development of case sites using PPR combined with key aspects of the research question highlights how utilisation of the described methodological approach may assist in exploring local health service provision in rural communities. The research aims can then be explored within and between identified cases relevant to the research. This is believed to be the first study to utilise this methodical approach to define rurality or relational place.

### Research method

The investigation site was a large area of one Australian state with a mix of rural, regional and remote communities with a range of health services of varying sizes. The scope of the study was bounded by the defined geographic area of a rural health service network. This area offered the opportunity to explore rural physiotherapy services across many health service settings. Identification of multiple sites enabled development of rural physiotherapy cases for exploring rural physiotherapy service provision. Ethics approval was obtained from the Human Research Ethics Committee of both James Cook University (approval number H3799) and the health service of the study. Data collection occurred from January to September 2012. Site specific approval was obtained for 25 health facilities employing physiotherapists within the investigation site.

Public sector physiotherapy service provision formed the primary focus of this research. The researchers’ experience as rural physiotherapists and reports of a greater reliance on the public sector for the provision of allied health services, including physiotherapy, in rural and remote regions of Australia [31] informed this focus. Private physiotherapy services, while limited in some communities, deliver significant services and formed a second focus of this investigation.

A preliminary quantitative component, a survey of public sector physiotherapists in the selected investigation area, preceded and guided the main qualitative data collection by informing purposive sampling and establishing preliminary results for further in-depth exploration [9]. Public physiotherapists were invited to participate in the initial phase of the research through local professional networks. Senior physiotherapists in the investigation site provided key physiotherapy contacts in the public sector physiotherapy departments. Surveys were mailed to the key physiotherapy contacts for distribution to colleagues in their facility. Participant information and consent forms were included with the survey for distribution. Physiotherapists were invited to complete a survey describing their setting, service and factors influencing their practice.

Results from the survey assisted in identifying a range of stakeholders willing to participate in the second stage of the study. Stakeholders included physiotherapists, their colleagues, managers, key decision-makers and consumers. The second stage of data collection consisted of stakeholder specific surveys. To obtain input from stakeholders, case site physiotherapists who had agreed to participate in follow up interviews, were asked to distribute questionnaires to the stakeholders at their site, including managers, consumers and team members [32]. The physiotherapists were able to guide or direct this data collection phase to stakeholders they identified as relevant to the decision-making process. Where present, the private physiotherapists were identified from listings in the Yellow Pages phone book of each case site. As the physiotherapy workforce has become increasingly privatised [33] it was important to consider the contributions to rural physiotherapy service provision made by private physiotherapists. To allow for possible intersectoral comparison of responses from consumer surveys, outpatient services were prioritised in the public health services. Both public and private physiotherapists were asked to place consumer surveys on relevant reception desks with an information sheet describing the study to allow consumers to elect to participate or not. Written consent was obtained from all participants. All surveys were anonymous unless participants agreed to provide their details for subsequent interviews.

In the final stage of data collection the principal researcher conducted face to face semi-structured interviews with a purposive sample of physiotherapists and key decision-makers. The interview questions reflected both the questions and responses of the survey as the researchers sought to obtain greater insight into the issues raised. The interviews occurred in each case site at a location and time convenient to each participant. Written informed consent was gained prior to the interview. All interview participants were informed that they would not be identifiable and that confidentiality would be maintained. This staged approach to data collection is consistent with the funnel analogy described by Bogdan and Biklen [34]. Interviews were ceased when data saturation occurred.

### Data analysis

Initial data analysis of the survey data was undertaken using Microsoft Excel spread sheets. Responses to open ended questions informed the development of initial themes and areas for further exploration in interviews. Manual and electronic recording of data through the use of NVivo version 10 were used to organise the qualitative data. Each interview was recorded and transcribed verbatim and entered into NVivo. Full interview transcripts and a summary developed by the researcher were provided to interview participants for their review and comment. An iterative approach was used to guide the qualitative data analysis [35]. The principal researcher completed the initial analysis with co-researchers double coding one third of the interviews to add to the depth of analysis. Thematic analysis was undertaken to develop tentative themes and concepts to develop codes, which were then used to frame and account for the remaining data [35].

### Research rigour

The research design included data collection from multiple sources to enable triangulation of data and constant comparison. Interviews were audio taped and transcribed verbatim with full transcripts and summaries provided to each interview participant for verification and additional comments. One third of the interviews (7/19) were coded by a second coder to verify themes. An auditable trail of evidence was maintained throughout the conduct of the research to further add to the credibility of the findings [15,35,36].

## Results

Physiotherapy surveys were received from 11 of the 25 (44%) public sector facilities identified as providing physiotherapy services in the investigation area. The sixteen completed surveys received from the 11 sites represented a 29.4% response rate as 54 physiotherapy surveys were distributed. From the surveys a matrix was developed to identify cases for purposeful sampling [35]. The surveys identified two key factors relevant to rural physiotherapy service provision: rurality and the number of physiotherapists. In view of the expressed limitations of geographic classification systems [30] participant perspectives of rurality (rural, regional or remote) were used to inform cases. The number of co-located colleagues was the second factor identified as a potential differentiating factor of rural physiotherapy service provision. This is consistent with the literature in which workforce and position shortages are recognised as characteristics of rural physiotherapy [37].

An example of an stratified purposive sampling [6], the proposed matrix had a potential total of 12 cells although many may not be applicable (Table 1). A regional setting with only a part time physiotherapist (less than one Full Time Equivalent (FTE)) is one such example. In addition to informing case site selection, participant perspectives of rurality were compared to current rural classification systems to identify commonalities and differences. Interview responses of participants at identified case sites then further contributed to concepts of rurality relevant to rural physiotherapy service provision.

**Table 1:** An initial matrix

	**≤1 Physio**	**1-2 Physios**	**3-5 Physios**	**>5 physios**
Remote				
Rural				
Regional				

The initial matrix was revised to reflect stakeholder responses including the larger referral centres and mixed stakeholder responses about the rurality of one location (Table 2). Six case types emerging from the physiotherapy responses. A further 23 surveys (five private practitioner, 13 colleague/manager and five consumer) were received from stakeholders at identified case sites. Nineteen interviews were conducted across the sites of the study.

**Table 2:** Revised matrix

**Physio FTE**	**≤1**	**2-3**	**4-10**	**>10**
Remote				
Rural- remote			7	
Rural	3 6* 5 9^ 11*	1, 10^	4	
Regional			8	2

^*services provided by the same physiotherapist.

Participant responses highlight the conceptual challenges when describing rurality and defining regional, rural and remote. For example participants who worked in a location with more than ten FTE physiotherapists stated:

***“*** *I suppose regional, yes. I don’t really consider myself to be rural. For me it is, I don’t consider this to be rural just because I can live here and have a city lifestyle without the stress and the traffic and the pollution. I don’t believe I’m living the rural lifestyle. If I had a rural lifestyle I’d have a farm”. [A3]*

***“*** *I suppose we’d be regional, I think of myself as rural but I think it’s probably regional”. [D1]*

One participant who worked in an area with between four to ten FTE physiotherapists stated that their perception of rurality was to some degree based on patient location.

“And that was the big thing from city versus country physio or rural physio, a lot of my patients travel six hours to see me”. [B4]

Participants also felt that access to services also assisted in defining the rurality of a location. For example a participant from a large country centre noted: *“the capacity to access high level services is limited* [here]*…and so the capacity to access the higher level service I think is one of the things that defines this as remote”. [D4]*. This was also reflected in smaller rural locations, adding to the notion of access to services and support as an important consideration in understanding rurality.

“The differences are like in metropolitan – in provincial … we've now got some specialists in most places. Whereas here you're expected to know hands and everything else so at least you’ve got a context. So it’s a video conference with the people for hands in [the capital city] but getting into the video conferences is an issue because that’s in the hold and treat rooms [for mental health patients]”. [A8]

## Discussion

The rurality of the case types reflected the way in which physiotherapists identified the setting in which they practice. Physiotherapy participants described eight of the eleven sites as rural, two as regional and one as remote-rural (Table 2). Fulltime equivalent (FTE) physiotherapist numbers ranged from 0.4 FTE (i.e. one physiotherapist working two days per week) to 14 FTE across the sites of this study. Six case types emerged from the responses from the public sector physiotherapists. Physiotherapist perception of rurality (PPR) in sites with four to ten physiotherapists was an important factor in making distinctions between sites, whereas the number of FTE physiotherapists was a greater differentiator in rural and regional sites. Fulltime equivalent categories could be further differentiated, but for the purpose of this study four categories were used.

Fewer case types would have emerged if a single measure of rurality was the only differentiating factor. Three case types would emerge if PPR, RRMA or ARIA were used as a single measure and only two case types if ASGC was to be used as the only differentiating factor. Comparison of rurality for each site using remoteness classifications revealed a variable picture (Table 3). The differentiated case types that emerged from the dual measures of PPR and FTE informed the selection of cases for this study.

**Table 3:** Comparison of cases using different rurality classification systems

**Sites**	**1**	**2**	**3**	**4**	**5**	**6**	**7**	**8**	**9**	**10**	**11**
ASGC-RA	3	2	3	2	3	2	3	2	3	2	3
RRMA	5	3	5	3	5	5	4	4	5	3	5
ARIA	A	HA	MA	A	MA	A	A	HA	A	A	A
PPR	Rural	Regional	Rural	Rural	Rural	Rural	Remote-Rural	Regional	Rural	Rural	Rural
Population (,000)	10-20	35-45	1-5	35-45	1-5	1-5	15-25	30-40	<1	5-10	<1
Public HHS beds	<50	200-500	<50	100-200	<50	<50	50-100	50-100	<50	<50	0

Australian Standard Geographical Classification Remoteness Areas [ASGC- RA]. Access/Remoteness Index for Australia [ARIA]. Rural, Remote & Metropolitan Areas Classification [RRMA]. Participant Perception of Rurality [PPR]. HA: highly accessible, A: Accessible, MA: moderately accessible.

The complexity of understanding the concept of rurality was revealed by interview participants when discussing perceptions of rurality relevant to their setting. Issues around the concept of rurality include the following:

• Is it the practitioner or the setting that defines rural health;

• is it about distance from a centre or a service provider;

• is it service size including the number of providers or a sole part time worker;

• is it about workforce availability;

• is it about the type of work undertaken such as specialist or generalist skills;

• is it about support available to the health professional;

• is it being visible and accountable to the community;

• is it about local knowledge or

• is it about distance from decision-makers?

Questions such as these reveal the convolutions of rurality and the variability often reported in the literature around rural health service provision [31,38,39]. Such variation further reinforces the need for the development of measures that can reflect this complexity and variation. Use of only a geographical classification of rurality is not sufficient to be able to distinguish between sites and thus cases when undertaking rural health service research. Dual measures, such as rurality and workforce numbers, provide more relevant differentiation than a single measure of rurality as defined by geographic classification. Similarly the continued use of catch-all term such as ‘rural health’ can limit the understanding of the similarities and differences found across locations [40]. Without understanding the associations between the specifics and context of each place, the attributes within the population and individual health services being delivered there is a large gap regarding the understanding of the specifics of health services in local rural communities.

This study adds to the literature describing limitations in the application of geographical classifications for differentiating rural health services. This study revealed that participant perspective of rurality often differed with the geographical classification of their location. For example, one participant expressed a sense of isolation more consistent with remote areas than that of a rural location.

“*in Katherine and things like that, in what's considered remote area and yet* [here] *I'm the only Allied Health therapist…so I'm actually probably.... professionally more isolated than a lot of these people …at least have teams in more remote areas”. [A8]*

Not only does the health professional feel isolated but access to services would be likely to be a key issue for residents in this location. This is consistent with the work of McGrail and Humphreys [30] which suggests that access to health services is a function of several factors including availability, proximity, health needs and mobility. The effect of distance on the accessibility to health care services has been identified as a key factor differentiating rural and remote from metropolitan health care [38]. Population size and geographical location then influence the mode and form of service delivery with socio-economic and geographic inequities influencing access to health care [38]. Implications for provision of health care, particularly primary health care have been discussed in the literature [38] however implications for provision of specific health services such as physiotherapy in rural settings are less evident.

Variability and diversity are characteristic of rural Australia [7], and combine with the unique demography of rural and remote Australia as key determinants of health problems and health service needs [8]. Adopting an approach that enables insight into variability and diversity of different sites is consistent with the dynamic characteristics of place. Health service provision in rural areas is increasingly influenced by networks, connections and linkages that may occur within defined boundaries of a local health system, but are increasingly Examples that emerged in this study include the impact on smaller rural physiotherapy service providers when new regional services are established and how a national decision such as activity based funding influences the health system and service delivery at all levels.

"recently I have told by [the regional centre] that I need to do all these lymphoedema patients and this is the pre assessment in terms of the all the whole population of anyone having breast cancer… to see them all – measure them all up before surgery… I said “I can’t do that” but that’s what I would be expected to do so, that is a direction coming from [the regional centre who are] saying we can’t do all of these". [A5]

"they just send them anywhere they can to get them out of [the regional hospital]…they have to get them out of there… you know within four days....[because of] funding, pressure, bed block … pressure to get them out, get them going, send them home". [A8]

*"[Length of stay]…it’s a* huge *measure and that, with activity based funding, they are going to be huge drivers.... Bed block, length of stay, money, will always stick up in their head and that is the thing that they will see as important* ***…*** *[the national health reform], it sort of sets the big agenda that will trickle down to us in other ways as in if they’ve got a project that they want that has a bucket of money, that will influence some decisions about what services are provided with that bucket of money and it just depends whether we’re in the mix or up in their face or not. Yes, it sets that whole funding agenda that’s going to have a major influence". [A1]*

These influences affect health service provision and can be lost when investigating service level decision making in rural health. The combination of missed methods utilising a survey followed by a collective case study and systems theory approach has demonstrated an appropriate framework to identify the issues surrounding rurality. Without further understanding of rurality, investigation into rural health will be lacking. Utilisation of this mixed methods approach could be applied to other rural health issues and may help to add to the research around health service delivery.

### Limitations

The research framework described was applied to an investigation of physiotherapy service provision in one area within Australia which had a mixture of remote, rural and regional centres. The framework and results may not be applicable across professions or to other areas with a different mixture of services or locations. Participant perception of rurality and public sector staffing numbers, while relevant to this study to define cases, may not be applicable to other studies.

## Conclusion

The framework described provides a structure to gain insight into local health service provision in a way existing statistical data does not. The combination of the systems theory-case study heuristic and a sequential mixed methods approach supported a qualitative exploration of issues identified through initial surveys. Obtaining participant perspectives of issues of interest, such as rurality and physiotherapy service provision, provides local detail often not available or visible. The concept of relational place aligns well to both systems theory and case study to aid exploration of the specifics of rural communities and the impact of context [1]*.* Concepts such as rurality and place then inform exploration of a health care issue of interest within and between rural communities. Defining key aspects of the research, such as participant perspectives of rurality and physiotherapy Full Time Equivalent, assist to define cases in which to explore the issue of interest. Adopting a systems approach then allows description of interrelated elements in each individual case while recognising interaction between cases and within the larger health systems.

## Abbreviations

ARIA: Access/remoteness index for Australia; AIHW: Australian institute of health and welfare; ASGC- RA: Australian standard geographical classification remoteness areas; FTE: Full time equivalent; PPR: Participant perception of rurality; RRMA: Rural, remote & metropolitan areas classification.

## Competing interests

The authors declare that they have no competing interests.

## Authors’ contributions

RA: was involved in concept development, study design, data collection, data analysis and interpretation and drafting of the manuscript. AJ: was involved in study design, data analysis and interpretation and drafting the manuscript. SL: was involved in study design, data analysis and interpretation and drafting the manuscript. LS: was involved in study design, data analysis and interpretation and drafting the manuscript. All authors read and approved the final manuscript.

## Pre-publication history

The pre-publication history for this paper can be accessed here:

http://www.biomedcentral.com/1471-2288/14/94/prepub
